# Toxicity of Flare and Crude Hydrocarbon Mixtures

**DOI:** 10.1100/tsw.2002.220

**Published:** 2002-05-22

**Authors:** Sonja V. Cook, Angus Chu, Ron Goodman

**Affiliations:** Department of Civil Engineering, University of Calgary, 2500 University Dr. NW, Calgary, Alberta, Canada T2N 1N4,

**Keywords:** hydrocarbons, hydrocarbon mixtures, toxicity, saturates, aromatics, asphaltenes, polars, bioavailability, fractionation, body burden, lethal body burden, narcosis, crude oil, flare pits

## Abstract

The toxicity of whole, saturate, and aromatic hydrocarbon mixtures from flare pit and crude oil sources were evaluated using *Lumbricus terrestris*. Body burden analysis was used to analyze the intrinsic toxicity of the six hydrocarbon mixtures. The major fractions of the whole mixtures, the saturate, and aromatic fractions had different intrinsic toxicities; the aromatics were more toxic than the saturates. The toxicity of the saturate and aromatic fractions also differed between the mixtures. The flare saturate mixture was more toxic than the crude saturate mixture, while the crude aromatic mixture was more toxic than the flare aromatic mixture. The most dramatic difference in toxicity of the two sources was between the flare whole and crude whole mixtures. The crude whole mixture was very toxic; the toxicity of this mixture reflected the toxicity of the crude aromatic fraction. However, the flare whole mixture was not toxic, due to a lack of partitioning from the whole mixture into the lipid membrane of the exposed worms. This lack of partitioning appears to be related to the relatively high concentrations of asphaltenes and polar compounds in the flare pit whole mixture.

## INTRODUCTION

Site assessment involving petroleum contamination typically involves the use of a generic soil guideline that establishes a soil hydrocarbon limit based on a composite analysis, such as total petroleum hydrocarbons. Generic limits for hydrocarbon contamination are meant to protect human and environmental heath[1], but are based on very limited toxicity data[2]. Further toxicological analysis of hydrocarbon mixtures is necessary and should include weathered mixtures as well as fresh hydrocarbon sources, since many hydrocarbon-contaminated sites involve weathered hydrocarbons[2]. The toxicological properties of the saturate and aromatic fractions of complex hydrocarbon mixtures should also be evaluated, since these fractions have distinct partitioning properties in the environment, and should ideally be kept separate in risk assessment of soil[3].

The toxicity of organic compounds and mixtures that act by narcosis can be evaluated by the amount of contaminant within the organism (body burden [BB]) that is associated with a toxic effect[4,5]. Assessment of the BB associated with a toxic effect, such as death, can be used to assess the toxicity of soil hydrocarbon contamination, without considering all of the kinetic and partitioning effects in the soil system, and should provide a better estimate of the mortality dose than exposure soil concentrations[4,6,7,8]. The lethal body burden (LBB) should be similar for organic chemicals with the same mode of action, relatively constant among species, and independent of exposure medium[8]. Determination of LBBs for saturate, aromatic, and whole mixtures of weathered flare and crude oil hydrocarbon sources will allow the assessment of the effective and intrinsic toxicities of these mixtures.

## EXPERIMENTAL METHOD

Hydrocarbons (oil and grease) were extracted from a flare pit soil using dichloromethane (DCM) Soxhlet extraction. Flare pits are earthen pits used to contain liquid wastes and produced water from the processing of natural gas and crude oil, as well as for flaring produced gas[9,10,11,12]. The soil in and around these pits is typically contaminated with medium- to heavy-molecular-weight hydrocarbon residues and pyrolized hydrocarbons, as well as high levels of metals and salts[9,10,13]. As a second hydrocarbon source, 20 l of topped Federated crude oil (for properties, see Bobra[14]) were used.

The oil and grease extract from the flare pit soil and the whole topped crude oil mixture were separated into maltene and asphaltene fractions by a pentane precipitation method adapted from Whittaker et al.[15]. Fraction separation of the flare and crude maltene mixtures was performed on silica gel columns[16,17]. Eluted fractions from multiple separations were combined and concentrated to provide stock saturate and aromatic samples for toxicological analysis.

Six contaminated soils were made using the whole flare (oil and grease extract), whole topped crude, flare saturate, crude saturate, flare aromatic, and crude aromatic hydrocarbon mixtures. Each contaminated soil was made by adding the hydrocarbon mixture to artificial soil (70% sand, 20% kaolinite clay, 10% peat by weight) to a final concentration of 30% hydrocarbons (by weight).

Toxicological analysis of the contaminated soils was performed using a 28-day earthworm mortality test using *Lumbricus terrestris*. *L. terrestris* were purchased from Carolina Biological Supply (Burlington, NC), placed in commercially available bedding (Magic Worm Ranch), and fed commercially available food (Magic Worm Food). The earthworms were stored at 5°C prior to use in the toxicity tests, and all toxicity tests were performed at 5°C. All earthworms were depurated for 48 h prior to the start of the toxicity experiments. The test soil (50 g) was placed in a 250-ml glass jar, and the soil was wetted to field capacity with distilled, deionised water. Four worms were placed in each jar. The toxicity of the six hydrocarbon-contaminated soils and control artificial soil were tested in triplicate. The worms exposed to artificial soil were used as controls for both the toxicity tests and BB analysis. Skin tissue of all earthworms that died during the test, and the live worms remaining after the 28-day exposure period were analysed for hydrocarbon BB. Skin tissue samples were extracted by grinding with DCM; the DCM extracts were cleaned up using silica gel cartridges. The extracts were analysed by GC-FID (HP6890 GC-FID with a HP6890 series Automatic Sampler). HP-5MS low bleed columns, 30 m × 0.25 mm, with a 0.25 mm silicone-coated fused-silica capillary column were used.

Solvent-extractable hydrocarbons were quantitated by GC-FID for each carbon number from C12 to C31. This carbon range includes the carbon range for the hydrocarbons exposed to the earthworms, but excludes the range of C31+, which contains large peaks of unidentified, unresolved earthworm tissue extracts in control and experimental samples. Surrogate recovery (*p*-Terphenyl-$d_{14}$) of individual samples was used to correct for sample loss during analysis. Samples with surrogate recoveries outside 99% warning limits were considered corrupted and excluded from the analysis. Because the DCM extract of the skin tissue sample contains all of the organic solvent–soluble components of the worm, the DCM extract weight is equivalent to the lipid weight of the sample earthworm. All body burdens (BBs) are therefore reported as mg/kg DCM extract weight (mg/kg ex wt), which is equivalent to the lipid-normalised earthworm BB.

Mean LBB and sublethal BB of live worms were determined for all worms in each treatment group. BB values of live worms and dead worms from the same exposure treatment were kept separate for statistical comparison. Control-corrected LBBs and BBs were determined by subtracting the mean BB of the control worms; control correction was only performed for group averages. The control-corrected LBB and BB can be assumed to exclude any solvent-extractable tissue from the worm tissue itself and to, therefore, represent the mean amount of petroleum hydrocarbon present in the lipid membrane for each exposure treatment. The influence of hydrocarbon exposure on BB concentrations was determined by Analysis of Variance (ANOVA) at a = 0.05[18]. Tukey's Pairwise Comparison was used to determine which groups of data differed when the ANOVA result was significant and more then two groups were compared[19].

## RESULTS AND DISCUSSION

Carbon number profiles and fractional analysis of the flare and whole mixtures are shown in Fig. 1 and Fig. 2. The flare and crude whole mixtures were both unimodal over the C12 to C31 range. The crude whole mixture has higher relative abundance in the C12 to C19 range, but the flare whole mixture had a higher relative abundance in the C20 to C31 range. The flare and crude whole mixtures were similar in that saturates and aromatics were the most abundant fractions in both mixtures. The abundance of hydrocarbon polars was also similar in both mixtures. However, both the nonhydrocarbon polars and asphaltenes were at least ten times more abundant in the flare whole mixture than in the crude whole mixture.

The flare whole soil and crude saturate soils were nontoxic to earthworms; flare saturate soil was slightly toxic (see Table 1). The flare aromatic, crude aromatic and crude whole soils were very toxic, causing 100% mortality in all soil replicates. The time to 100% mortality was 7 days for the crude whole soil, 10 days for the crude aromatic soil, and 16 days for the flare aromatic soil.

The average (control-corrected) LBBs of the earthworms exposed to the hydrocarbon mixtures are shown in Table 2.

ANOVA analysis found no significant differences in the BBs between the earthworms exposed to the flare whole soil and the control earthworms, which were not exposed to hydrocarbons ($p > 0.05$). This result indicated that the partitioning of hydrocarbons from the flare soil into the tissue of the exposed earthworms was very low; not enough hydrocarbon was present at the membrane level to cause narcosis. ANOVA analysis was performed to determine if the LBBs for the other hydrocarbon exposure groups earthworms differed (see Table 3).

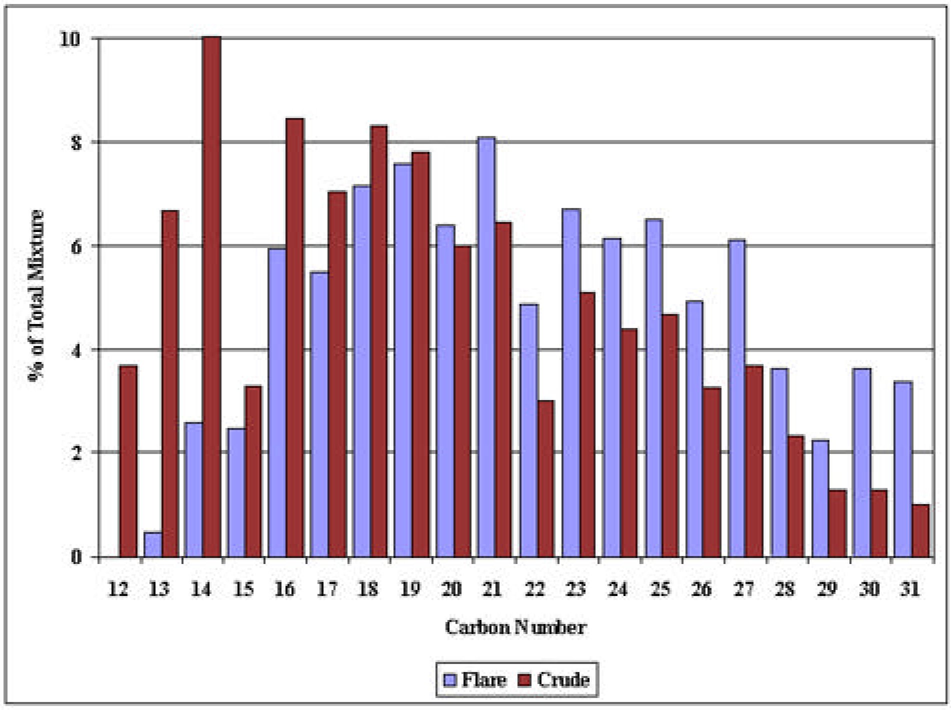

**FIGURE 1**. Carbon number distribution for flare and crude mixtures.

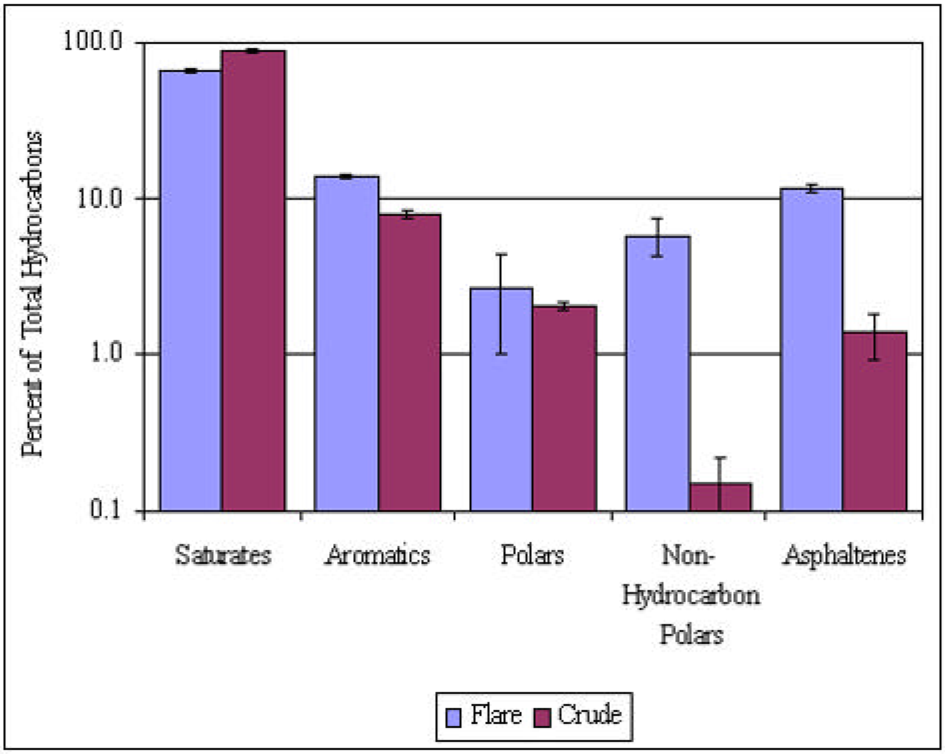

**FIGURE 2**. Hydrocarbon type distribution of crude whole and flare whole sources.

**TABLE 1**
**Toxicity of Flare and Crude Hydrocarbon Mixtures**

| Toxicity | | |
|---|---|---|
| **Non-Toxic** | **Slightly Toxic** | **Very Toxic** |
| Flare Whole | Flare Saturate | Flare Aromatic |
| Crude Saturate | | Crude Aromatic |
| | | Crude Whole |

**TABLE 2**
**Average LBB of Hydrocarbon Exposed Earthworms**

| Hydrocarbon Mixture | Average Lethal Body Burden (mg/kg) | SE |
|---|---|---|
| Flare Whole | — | — |
| Flare Saturate | 1670 | 625 |
| Flare Aromatic | 834 | 164 |
| Crude Whole | 865 | 222 |
| Crude Saturate | 1856 | 583 |
| Crude Aromatic | 371 | 81 |

**TABLE 3**
**LBB ANOVA Comparisons**

| Hydrocarbon Mixtures Compared | Significant Difference | Significantly Different Groups |
|---|---|---|
| Crude Saturates, Flare Saturates | No | None |
| Crude Aromatics, Flare Aromatics | Yes | Crude < Flare |
| Crude Whole, Flare Whole | No Comparison | |
| Flare Aromatics, Flare Saturates | No | None |
| Crude Aromatics, Crude Saturate, Crude Whole | Yes | Aromatics < Whole, Saturates |

## Comparisons Between Fractions

The LBBs of the flare saturate and aromatic fraction exposed worms were not significantly different, indicating that these hydrocarbon fractions did not have different intrinsic toxicities. Since the flare whole exposed worms did not have significantly higher BB than the control, no ANOVA comparisons were made between the whole, saturate, and aromatic exposed worms. However, aromatic and saturate exposed worms had BBs higher than the whole exposed worms, since the BBs for these exposures were higher than the controls. The BBs of the aromatic and saturate exposed worms were also lethal; whereas the flare whole exposed worm BBs were not. These results indicate that there are toxic hydrocarbons present in the whole mixture, but the partitioning of these compounds is somehow reduced when the whole mixture is exposed to the worms.

The high sublethal BBs of the worms exposed to the crude saturate fraction indicate that the intrinsic toxicity of the crude saturate fraction was lower than the intrinsic toxicity of the crude aromatic fraction. The crude whole LBB was significantly higher than the LBB of the aromatic exposed worms. Given the nontoxicity of the crude saturate fraction, it is likely that the total BB of the whole mixture exposed earthworms was higher than that of the aromatic exposed worms due to the presence of saturated hydrocarbons as well as aromatic hydrocarbons, but only the aromatic fraction likely has a toxic effect in the whole exposed earthworms.

### Comparisons Between Sources

The average LBB of the flare aromatic exposed worms was significantly higher than that of the crude aromatic exposed worms, indicating that the crude aromatic fraction is more intrinsically toxic than the flare aromatic fraction. The total BBs of the crude and flare saturate exposed earthworms were not significantly different. However, since the crude saturated BBs were sublethal, and the flare saturate BBs were lethal, it is apparent that the flare saturate fraction is more intrinsically toxic than the crude saturate fraction. The difference in toxicity between the saturate fractions may be explained by biological transformation, which has been shown to increase toxicity of hydrocarbon components[20,21,22,23,24], and likely has occurred more extensively in the flare hydrocarbon mixture than in the crude hydrocarbon mixture[25].

The LBB of the flare whole exposed worms could not be included in the statistical comparison because so few worms died in any of the experiments. However, the very fact that 30% hydrocarbons by weight flare whole soil was nontoxic, and the very low BB of the earthworms exposed to the flare whole soil, indicate that the crude whole mixture is far more toxic than the flare whole mixture.

The reduced partitioning of the flare whole mixture compared to the crude whole mixture cannot be explained by differences in saturate and aromatic fraction toxicity. The saturate and aromatic fractions of the flare and crude whole mixtures are bioavailable when exposed alone. The aromatic fractions from both mixtures have similar intrinsic toxicities, and the flare saturate fraction appears to be more intrinsically toxic than the crude saturate fraction. Therefore, if the saturate and aromatic fractions are primarily responsible for the whole mixture toxicity, the toxicity of the flare whole mixture should be expected to be higher than that of the crude whole mixture. Dilution of the toxic compounds due to the presence of a mixture alone cannot be responsible for the observed reduced partitioning. It is therefore likely that the presence of the other compounds present in the flare whole mixture, and not in the crude whole mixture, reduces the cellular exposure of the toxic saturate and aromatic hydrocarbons to the worm.

It has been shown that the presence of high concentrations of asphaltenes and polars (resins) can cause reduced degradation of otherwise bioavailable compounds in crude oil mixtures[26,27,28,29]. Asphaltene and resin compounds are typical of heavy-end and weathered hydrocarbon mixtures[30,31,32] and can be produced by the biotransformation of hydrocarbons[11,20,26,33,34,35,36]. The flare whole mixture is much higher in asphaltene and polars than the crude whole mixture (see Fig. 2), and the crude whole mixture does not show this reduction in toxicity and BB. It is therefore likely that the reduced bioavailability of hydrocarbons in the flare whole mixture is due to the presence of the asphaltene and polar compounds, which somehow reduce the exposure and effective toxicity of the otherwise more mobile and bioavailable contaminants in the mixture. This is a logical extension of the observed literature results, since degradation rates and effective toxicity are related to bioavailability of compounds within the whole mixture.

## CONCLUSIONS

The results of the toxicity tests of all of the crude and flare whole and fraction hydrocarbon exposures were consistent with the theory of hydrocarbon narcosis. However, intrinsic toxicity differences are apparent between the aromatic and saturate fractions of both the crude and flare pit hydrocarbon mixtures. The aromatic fractions were more intrinsically toxic than the saturate fractions, requiring a lower BB to cause a lethal effect. The flare saturate fraction was more intrinsically toxic than the crude saturate fraction, possibly due to biotransformation of the constituent compounds. Ecotoxicological effects of hydrocarbon mixtures at contaminated sites will therefore be a product not only of the factors which influence the bioavailability and amount of hydrocarbons available for partitioning, but also of the intrinsic toxicity and relative amounts of the saturate and aromatic hydrocarbons in the mixture.

The flare and crude whole mixtures exhibited much different toxicological effects. The crude whole mixture was very toxic, and consistent with the toxicity of the crude aromatic fraction. However, the flare whole mixture contaminated soil was much less toxic than would be expected, given the observed toxicity of the flare saturate and aromatic fractions. The effective toxicity of the flare whole fraction was greatly inhibited, apparently by high abundances of asphaltenes and polars in the mixture, which interfered with the partitioning of toxic hydrocarbons into the lipid membrane. Since these compounds are produced by biotransformation of hydrocarbons, it is logical to assume that the relative abundance of these degradation resistant hydrocarbons will only increase with time at a flare pit site. Therefore, even without bioremediation treatment, it is unlikely that environmental and toxicological impact of the hydrocarbons at the flare pit site will increase over time.

## ACKNOWLEDGEMENTS

This research was supported by NSERC (Natural Sciences and Engineering Research Council) and Imperial Oil Resources. This paper was presented at the CSIC/ESF workshop Analysis, Toxicity and Biodegradation of Organic Pollutants in Groundwater from Contaminated Land, Landfills and Sediments, Barcelona, Spain, 8–10 November 2001.
